# The Structure and Thermal Properties of Solid Ternary Compounds Forming with Ca^2+^, Al^3+^ and Heptagluconate Ions †

**DOI:** 10.3390/molecules25204715

**Published:** 2020-10-14

**Authors:** Ákos Buckó, Zsolt Kása, Márton Szabados, Bence Kutus, Ottó Berkesi, Zoltán Kónya, Ákos Kukovecz, Pál Sipos, István Pálinkó

**Affiliations:** 1 Department of Inorganic and Analytical Chemistry, University of Szeged, Dóm sqr. 7, H-6720 Szeged, Hungary; bucko@chem.u-szeged.hu (Á.B.); kasa.zsolt15@gmail.com (Z.K.); sipos@chem.u-szeged.hu (P.S.); 2 Department of Organic Chemistry, University of Szeged, Dóm sqr. 8, H-6720 Szeged, Hungary; szabados.marton@chem.u-szeged.hu; 3 Department of Molecular Spectroscopy, Max Planck Institute for Polymer Research, D-55128 Mainz, Germany; kutusb@chem.u-szeged.hu; 4 Department of Physical Chemistry and Materials Science, University of Szeged, Rerrich B. sqr. 1, H-6720 Szeged, Hungary; oberkesi@chem.u-szeged.hu; 5 Department of Applied and Environmental Chemistry, University of Szeged, Rerrich B. sqr. 1, H-6720 Szeged, Hungary; konya@chem.u-szeged.hu (Z.K.); kakos@chem.u-szeged.hu (Á.K.)

**Keywords:** calcium, aluminate, Bayer process, heptagluconate, ternary compounds, structure of solid compounds

## Abstract

In the present work, the structure and thermal stability of Ca–Al mixed-metal compounds, relevant in the Bayer process as intermediates, have been investigated. X-ray diffraction (XRD) measurements revealed the amorphous morphology of the compounds, which was corroborated by SEM-EDX measurements. The results of ICP-OES and UV-Vis experiments suggested the formation of three possible ternary calcium aluminum heptagluconate (Ca-Al-Hpgl) compounds, with the formulae of CaAlHpgl(OH)_4_^0^, Ca_2_AlHpgl_2_(OH)_5_^0^ and Ca_3_Al_2_Hpgl_3_(OH)_9_^0^. Additional IR and Raman experiments revealed the centrally symmetric arrangement of heptagluconate around the metal ion. The increased thermal stability was demonstrated by thermal analysis of the solids and confirmed our findings.

## 1. Introduction

Bauxite ores are the primary raw materials of the large-scale production of alumina, due to their high aluminum content, availability, as well as the relative simplicity of their refinement. During the Bayer process, i.e., the recrystallization of the aluminum content of bauxite in the form of Al(OH)_3_, the causticity of the process liquor must be maximized to improve productivity. Therefore, lime (Ca(OH)_2_) is added to the spent liquor to convert the in situ formed sodium carbonate to sodium hydroxide via the precipitation of calcium carbonate. This so-called recausticization step is an integral part of most Bayer refineries operation; hence, lime belongs to the primary raw materials of alumina production, and is essential for the causticization process to be a cost-effective operation of a Bayer refinery [1]. As such, the chemistry of calcium hydroxide in Bayer liquors has attracted considerable scientific as well as technological interest [2,3,4].

In a recent review, Rosenberg et al. gave a detailed overview of the solid calcium- and aluminum-containing species relevant to the Bayer process. Accordingly, the most stable solid calcium species forming in sodium aluminate solutions is the Ca_3_Al_2_O_6_∙6H_2_O (tricalcium-aluminate hexahydrate, TCA) [5]. Additionally, they detected two metastable layered double hydroxides (LDHs), namely the [Ca_2_Al(OH)_6_]_2_∙1/2CO_3_∙OH∙nH_2_O and the [Ca_2_Al(OH)_6_]_2_∙CO_3_∙nH_2_O compounds. In these solids, the Al(OH)_6_ polyhedra are connected by Ca atoms whose coordination shell is completed by hydrating water molecules, as inferred from the X-ray diffraction (XRD) analysis of the chloride analogue, i.e., so-called Friedel’s salt [6]. The formation of LDHs is a critical reaction step in the recausticization of the liquor. Although these intermediate processes first result in a decrease in the concentration of both carbonate and aluminate ions, the latter is recovered due to the decomposition of LDHs above 80 °C, yielding calcium carbonate and soluble aluminate.

Apart from inorganic components, low-molecular-weight organic compounds are also introduced into the Bayer process by most bauxites. Since the accumulation of these materials over time may have an impact on the causticization process of the refineries, a deep understanding of the speciation behavior of calcium(II) in the presence of these organics is vital to predict the solubility of lime in Bayer liquors. In this respect, The and Sivakumar investigated the effect of organic molecules on the solubility of calcium(II) in strongly caustic liquors. They reported that humic acid and sodium gluconate (used as model compounds in their study) significantly enhanced the solubility of calcium(II) [7]. From these results, one can infer strong interactions between calcium (II) ions and these organic molecules in solution, that is, the formation of stable compounds.

**Scheme 1:**
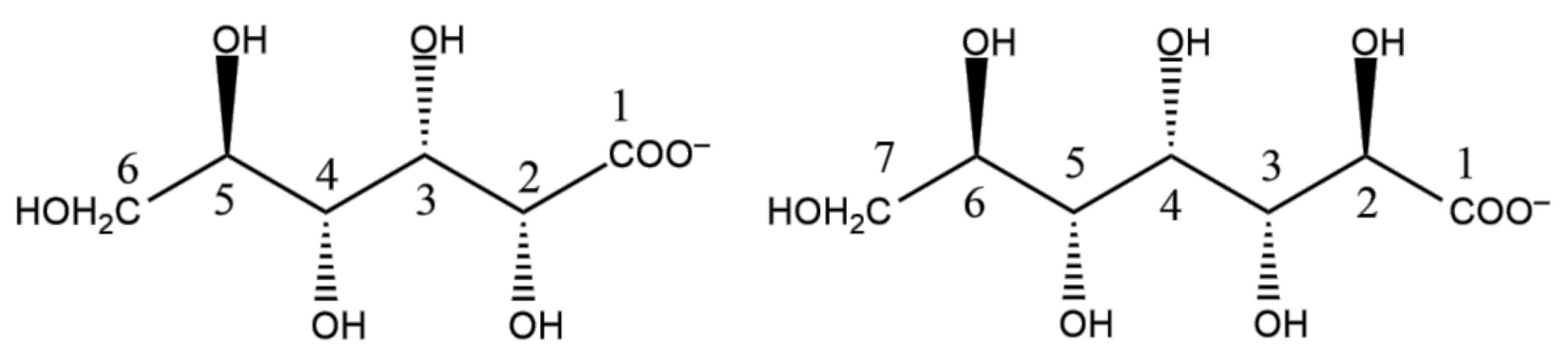
Structural formulae of d-gluconate (**left**) and d-heptagluconate (**right**) ions.

The solution equilibria between sugar-type ligands (such as gluconate, gulonate, heptagluconate, isosaccharinate) and Ca^2+^ or Al^3+^ ions in acidic to neutral medium have been by now well established [8,9,10,11,12,13,14]. Additionally, complexation reactions in strongly alkaline medium have been quantified and deep understanding on metal-ligand interactions have been gained [15,16,17]. That is, these complexing agents act exclusively as multidentate ligands via simultaneously binding metal ions by the COO^−^ as well as one or more OH or O^−^ functions, yielding stable chelate structures. On the other hand, little is known about the interactions in a three-component system containing both metal ions and a complexing agent. In particular, quantitative description of such systems is important, since, based on the results with other tri- or tetravalent cations reported in the literature [18,19,20,21], Ca^2+^ ions and sugar derivatives may form ternary compounds with Al^3+^ ions too. In this respect, for example, ternary species were proposed to form when gluconate (Scheme 1) was added to a suspension of tricalcium aluminate, based on qualitative X-ray diffraction and infrared analyses [22,23].

The working hypothesis of the current contribution is that in order to adequately describe the solution equilibria of such a ternary system, one has to consider the incidental formation of solid ternary compounds. In addition to this, characterization of the solids precipitating from binary solutions allows for a separate analysis of the interactions between the heptagluconate and one metal ion (Ca^2+^ or Al^3+^), which helps to disentangle the rather complex binding motifs that may be present both in ternary solids and solutions containing the constituents of the ternary compounds.

In this paper, we report on the qualitative and quantitative analysis of the solid phases forming in solutions containing calcium, aluminate and heptagluconate (Hpgl^−^) ions, with the latter serving as a model organic ligand (Scheme 1). A set of experimental methods were used, i.e., X-ray diffraction (XRD), (differential) thermogravimetry (TG/DTG), scanning electron microscopy coupled to energy-dispresive X-ray spectroscopy (SEM-EDX), Fourier-transform infrared (FT-IR) and Raman as well as inductively-coupled plasma optical emission spectroscopies (ICP-OES).

## 2. Results and Discussion

### 2.1. Crystallinity of the As-Prepared Binary and Ternary Samples as Well as Reference Compounds

**Figure 1:**
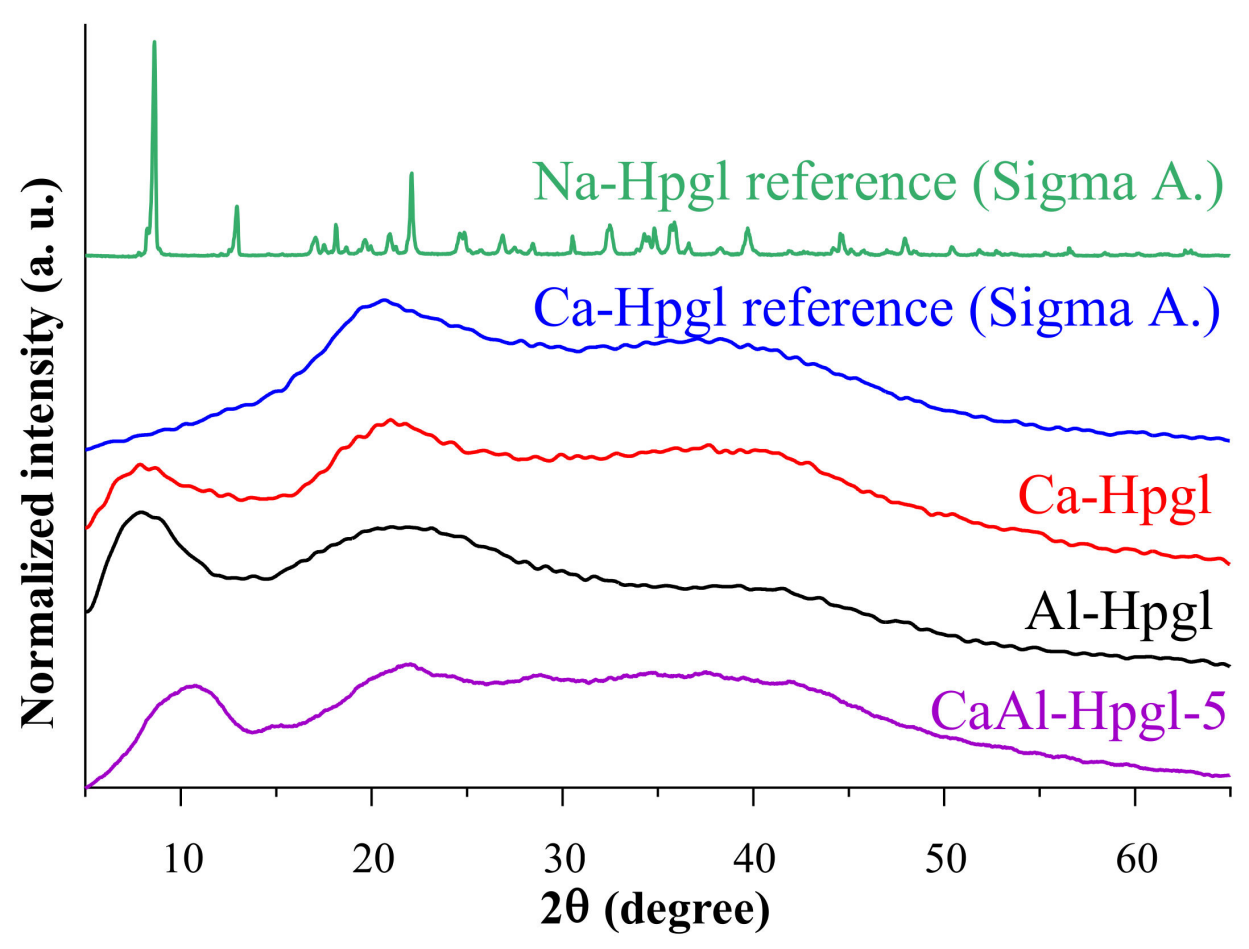
X-ray diffractograms of the reference heptagluconate (Hpgl^−^) salts, the as-prepared binary Ca- and Al-Hpgl solid compounds and a typical ternary Ca-Al-Hpgl compound.

First, the powder X-ray diffractograms of the precipitates as well as the Na^−^ and Ca^−^ heptagluconate reference compounds were recorded to gain information on the reflection patterns and crystallinity, respectively. While NaHpgl × nH_2_O has sharp and well-separated reflections (Figure 1), the diffractograms of the Ca(Hpgl)_2_ × 2H_2_O reference, the as-prepared binary Ca-Hpgl and Al-Hpgl samples are rather broad and not well-structured indicating that the binary compounds are amorphous. Similarly, the diffractogram of the ternary Ca-Al-Hpgl-5 sample shows broad reflections with poor signal-to-noise ratio (Figure 1; for the other ternary compounds, see Figure S1 in the Electronic Supplementary Materials, ESI), referring again to a low degree of crystallinity. This finding is in line with the formation of amorphous Ca-Al-gluconate solid phases, reported previously [22].

### 2.2. Morphologies of the Binary and Ternary Precipitates as well as Reference Solids

**Figure 2:**
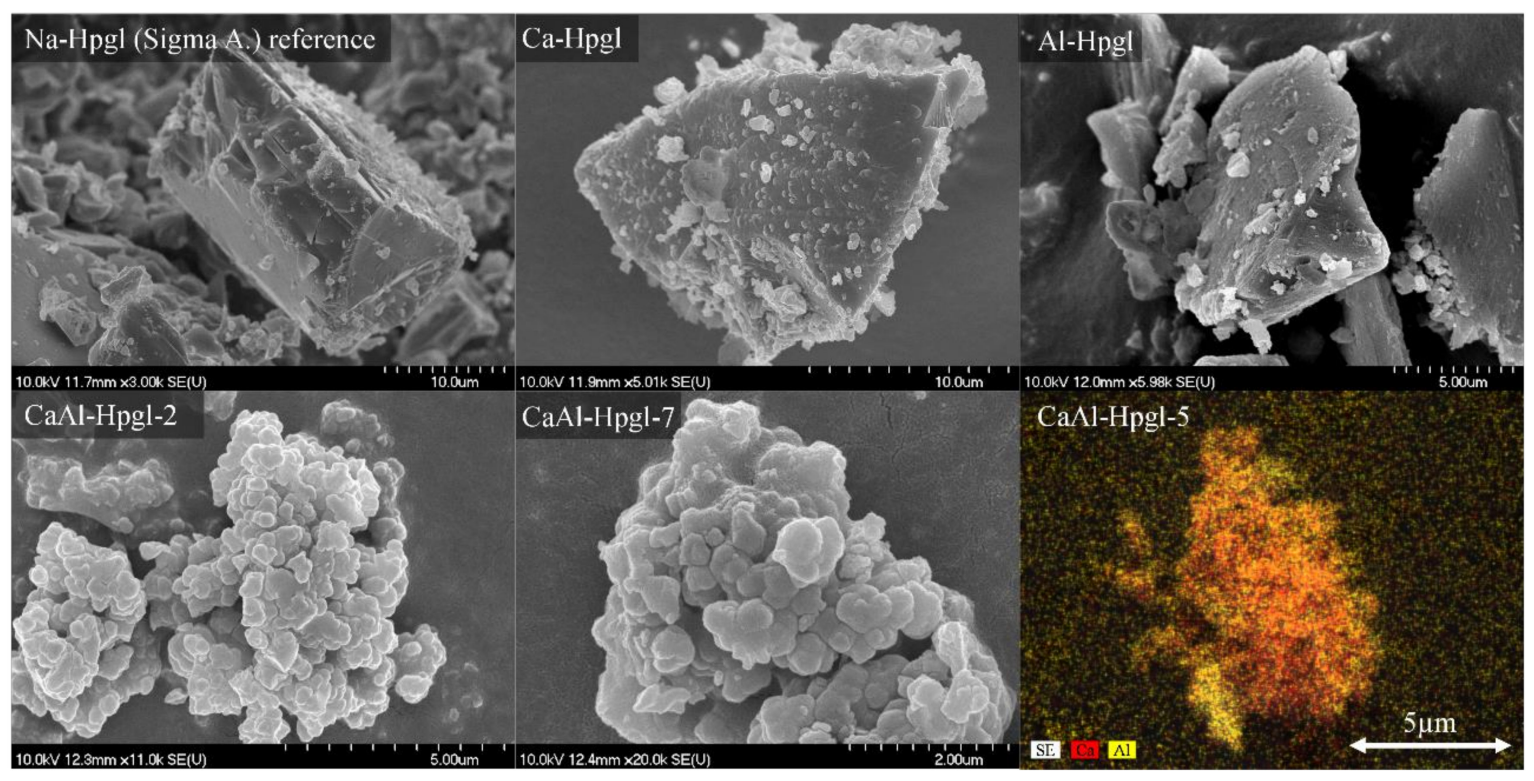
Scanning electron microscopy (SEM) images of sodium heptagluconate (Na-Hpgl) reference salt as well as the binary (Ca-Hpgl, Al-Hpgl) and ternary (CaAl-Hpgl) compounds with Ca^2+^ and Al^3+^ ions. The images of CaAl-Hpgl-2 and CaAl-Hpgl-7 represent the typical morphology, while the image of CaAl-Hpgl-5 represents the metal distribution of the ternary samples.

The results of the SEM analysis are in line with the findings of the X-ray diffractometry: compared to the angular particles of the well-crystallized heptagluconate sodium salt, the ternary and binary solids are rather amorphous, and they show significantly different morphologies (Figure 2). For all these samples, exclusively highly aggregated particles (in the range of 500–1000 nm merged into few micron sizes) with planar shape and smooth edges could be observed without the occurrence of crystallites. Meanwhile, the SEM-EDX analysis of the ternary samples (here CaAl-Hpgl-5 is shown in Figure 2) shows rather uniform distribution of calcium and aluminum atoms implying that this compound is indeed a ternary compound and not the heterogenous mixture of two binary solids (the separated elemental distribution mapping images can be found in Figure S2).

### 2.3. Component Quantification and Stochiometric Calculations

The total concentrations of Ca^2+^ and Al^3+^ for the binary and ternary samples (dissolved in ≈2 M HCl) were determined by ICP-OES, while the concentration of heptagluconate was estimated on the basis of UV-Vis spectrophotometry (see Section 3.3). The calculated mass percentages of Ca^2+^, Al^3+^ and Hpgl^−^ are listed in Table 1. The molar fraction of OH^−^ was determined applying the charge balance equation, and any residual mass was ascribed to the presence of surface or lattice water. The molar fractions of all components were referenced to that of Al^3+^ (which was set to unity), and the thus obtained cumulative stoichiometries are presented also in Table 1.

As a result, the Ca-Hpgl binary compound is most probably in the form of partially deprotonated CaHpgl_2_, while the formula of Al-Hpgl binary sample (AlHpgl_0.4_(OH)_2.6_) can be assigned to the trinuclear Al_3_Hpgl(OH)_8_^0^ compound, whose stoichiometry is similar to the previously reported Al_3_Hpgl(OH)_10_^2−^ species, which was found to be formed in strongly alkaline solutions [16].

In the systems containing Al^3+^, Hpgl^−^ and OH^−^ ions, where the predominant solution species are the AlHpgl_2_(OH)_5_^4−^ and AlHpgl(OH)_4_^2−^ at total concentrations given in Table 1 [16], the formation of the solid phase could be observed. As will be further discussed in Section 2.4, this may be attributed to the formation of at least one low-solubility ternary Ca-Al-Hpgl compound. Examining the calculated cumulative stoichiometry listed in Table 1, two types of compounds could be distinguished. In the first case, when the n_OH_/n_Hpgl_ ratio was above 1.0 (samples CaAl-Hpgl-1 to 4), the formation of CaAlHpgl(OH)_4_^0^ and Ca_2_AlHpgl_2_(OH)_5_^0^ could be suggested by the following reactions:

Ca^2+^ + AlHpgl(OH)_4_^2−^ = CaAlHpgl(OH)_4_^0^ (1)

2Ca^2+^ + AlHpgl_2_(OH)_5_^4−^ = Ca_2_AlHpgl_2_(OH)_5_^0^ (2)

The product forming in Equation (1) refers to the sample CaAl-Hpgl-1, while the stoichiometries of samples 2, 3 and 4 are interpretable by Equation (2). The excess Ca^2+^ and Hpgl^−^ might be assigned to binary Ca-Hpgl compounds, whose stoichiometry could not be defined unambigously.

On the other hand, for samples with n_OH_/n_Hpgl_ < 1.0, one major compound with a more sophisticated structure could be assumed. Examining the calculated stoichiometries for samples CaAl-Hpgl-5 to 8, the differences among them are only minor, which may refer the exclusive formation of only one compound, Ca_3_Al_2_Hpgl_3_(OH)_9_. Interestingly, the stoichiometry of the compound strongly resembles of that of TCA (Ca_3_Al_2_(OH)_12_), which may contribute to the understanding of the results published by Kim et al. [22,23].

Considering the average error of the obtained stoichiometric numbers (ν ≈ ±0.2) based on the assignation of Al-Hpgl, the proposal of the foregoing ternary species might be a good iteration of the calculated compositions.

### 2.4. The Effect of Metal Coordination on the Infrared and Raman Spectra of Sodium Heptagluconate

**Figure 3:**
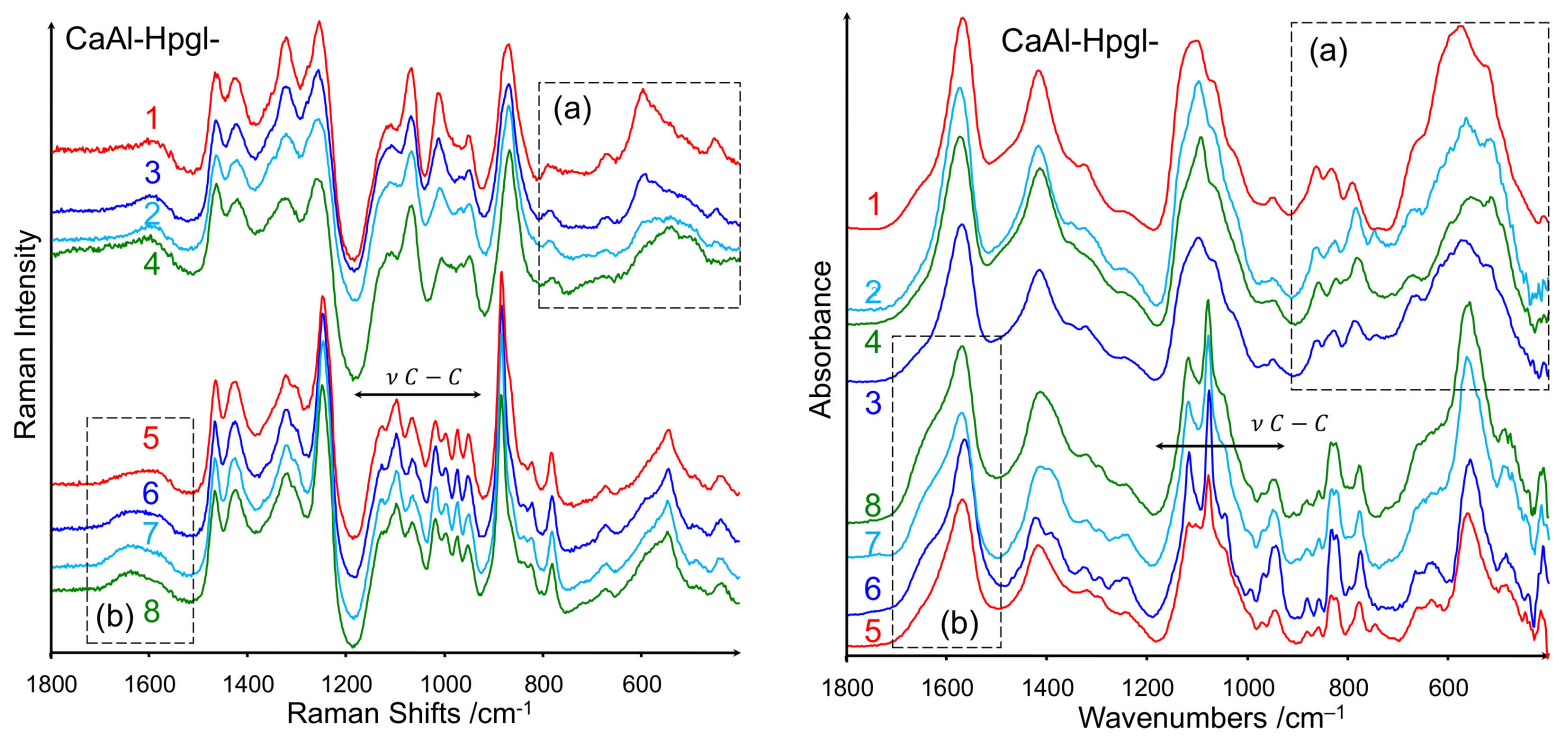
Raman and IR spectra of the ternary compounds. The difference between the C-C stretching region of the two groups indicates the difference in the conformation of the carbon chain (for the explanation of (a) and (b), see text).

The C-C stretching range of the Raman spectra divided the ternary samples into two groups showing clear difference in the conformation of the backbone of Hpgl^−^ (Figure 3). Raman spectra of samples from CaAl-Hpgl-1 to CaAl-Hpgl-4 differ only below 800 cm^−1^, in the out of plane deformation range of the carboxylate and C-H groups (Figure 3, left (a)). Only slight relative intensity differences can be identified elsewhere in the spectra.

The other group of samples from CaAl-Hpgl-5 to CaAl-Hpgl-8 produced much more uniform Raman spectra; they are almost indistinguishable, except around 1600 cm^−1^, in the carboxylate stretching region (Figure 3, left (b)).

The C-C stretching region of both groups differ essentially indicating the difference in the conformation of the carbon chain. This range is rather complicated in the spectra of CaAl-Hpgl-1 to CaAl-Hpgl-4, but the other set of spectra shows very clearly a series of nearly equidistant bands, the so-called “band progression,” i.e., the sign of a fully stretched carbon backbone [24].

The corresponding infrared spectra confirm the conclusions drawn from the Raman spectra (Figure 3, right). The magnitude of differences for both groups of spectra are essentially the same; therefore, the division of samples into two groups from CaAl-Hplg-1 to CaAl-Hplg-4 and from CaAl-Hplg-5 to CaAl-Hplg-8 is appropriate.

Another feature in the spectra worth mentioning is the carboxylate region, which can be seen in Figure S3b. The position of the peaks, tentatively assigned to the carboxylate stretching modes, did not coincide with the corresponding infrared and Raman spectra of the samples. Since the selection rules do not justify this difference, there were only two possibilities to take into account. One possible explanation is that the Hpgl is arranged in central symmetry around the metal ions resulting the separation of the “gerade” and “ungerade” species into the Raman and into the infrared spectra, respectively. On the other hand, the positions of the peaks could be the results of overlapping bands with various intensities, which is indicated by the differences in the carboxylate stretching region depicted in Figure 3 (b) [24].

A simple way to verify this assumption is performing Fourier Self-Deconvolution on the spectra between 1850 and 1200 cm^−1^. A typical result of the Fourier Self-Deconvolution is shown in Figure S4. The carboxylate stretching peaks split into at least two components in the infrared spectra. However, the deconvolution of the same range of the Raman spectra provided less information with the peak around 1600 cm^−1^ being too weak, and too broad for a successful Fourier Self-Deconvolution. On the other hand, the assumed symmetric carboxylate stretching band did not decompose.

**Figure 4:**
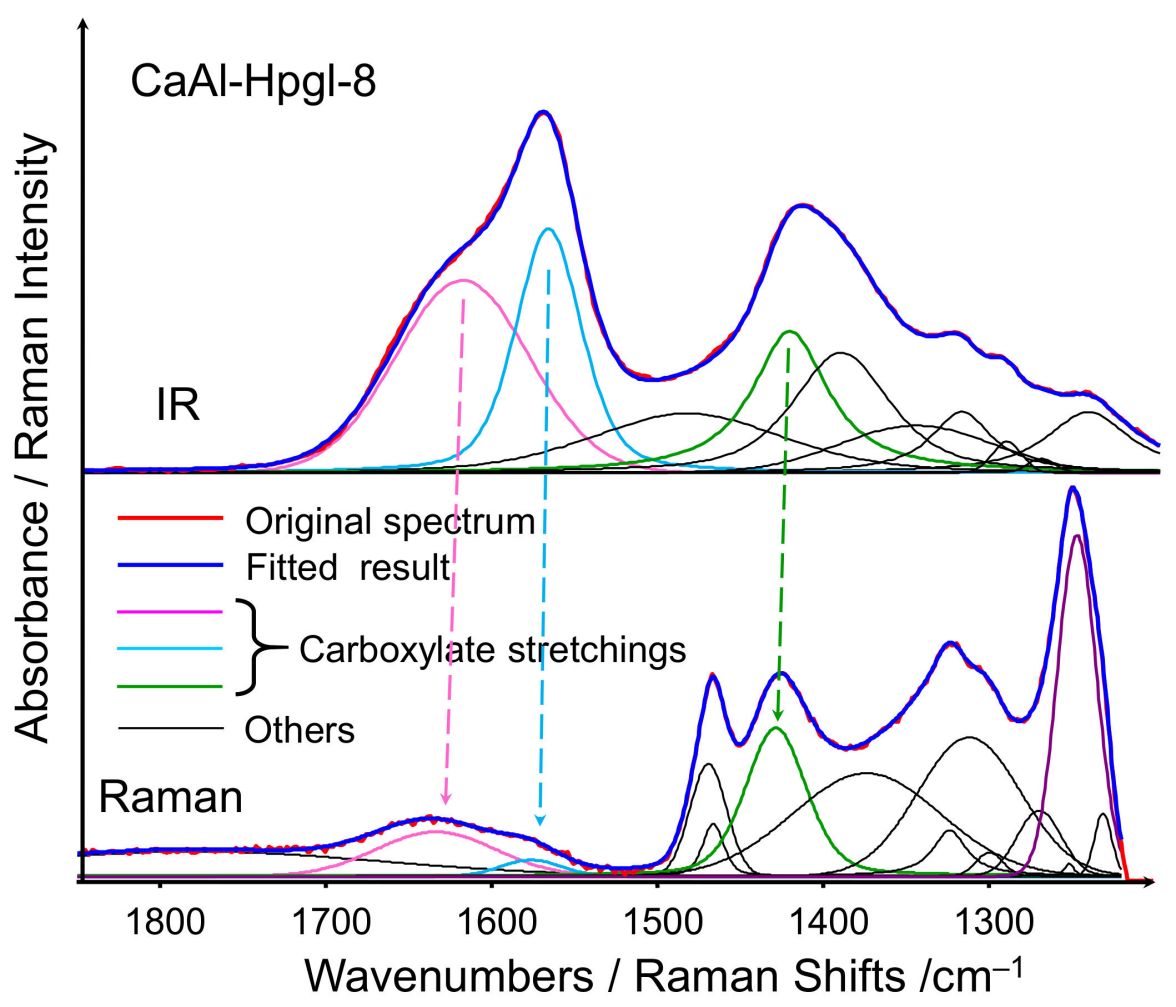
The result of peak fitting demonstrated on sample CaAl-Hpgl-8. The positions of the fitted carboxylate stretching bands, which refer to a symmetry center between the heptagluconate, are denoted by dotted lines.

In conjunction with Fourier Self-Deconvolution, peak fitting was performed with every spectrum in the same region. An example of the fitting process accomplished for sample CaAl-Hpgl-8 is depicted in Figure 4.

Concerning the fitting process, there were apparent deviations between the two groups of samples. Generally, fewer peaks were sufficient for the satisfactory fitting of the spectra of samples from CaAl-Hpgl-1 to CaAl-Hpgl-4 than for samples from CaAl-Hpgl-5 to CaAl-Hpgl-8. More importantly, no analogous bands could be found in the Raman spectra of the first group of samples between 1600 and 1500 cm^−1^ matching the strong infrared band of the antisymmetric carboxylate stretching mode. Moreover, the fitting of the Raman spectra would have been inadequate for the second set of samples without the corresponding, very low intensity bands. The positions of the fitted carboxylate stretching bands in the IR and the Raman spectra are satisfactorily close to each other ruling out the presence of a symmetry center between the Hpgl (for detailed data, see Table S1 in the ESI).

**Figure 5:**
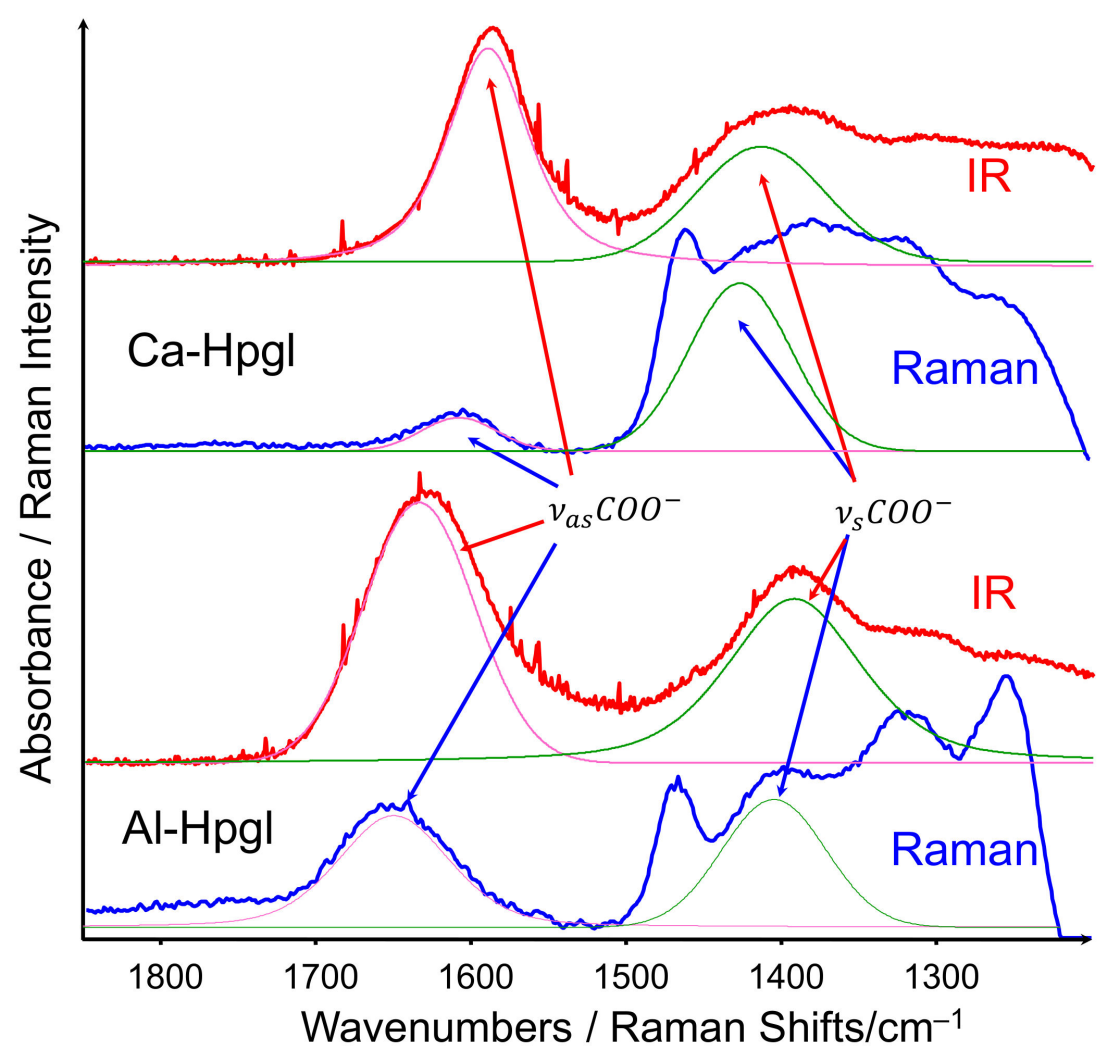
Fitting results of the Ca-Hpgl and Al-Hpgl binary samples. The antisymmentric carboxylate stretching mode of Hpgl^−^ above 1600 cm^−1^ refers to the coordination with Al(III), while between 1600 and 1500 cm^−1^ to that with Ca(II).

The spectra of the binary samples Ca-Hpgl and Al-Hpgl were also analyzed. Their spectra, with the carboxylate stretching bands are shown in Figure 5.

Comparing the fitted peak positions with those of the ternary compounds, one can infer that the presence of two bands in the antisymmetric carboxylate stretching region could be attributed to the coordination of Ca(II) and Al(III) ions to Hpgl^−^. Explicitly, the antisymmetric stretching mode above 1600 cm^−1^ corresponds to the coordination of Al(III), while the band between 1600 cm^−1^ and 1500 cm^−1^ may be the sign of coordination to Ca(II) [25,26,27].

Finally, the ratios of the integrated intensities of the fitted antisymmetric carboxylate bands above and below 1600 cm^−1^ corroborate the existence of the two distinct types of samples. The ratios were above 1.0 for the samples from CaAl-Hpgl-1 to CaAl-Hpgl-4, and below 1.0 for the rest, which indicates lower amounts of Al(III) coordinated to Hpgl^−^ in the first group (Table S1).

### 2.5. Thermal Analysis

**Figure 6:**
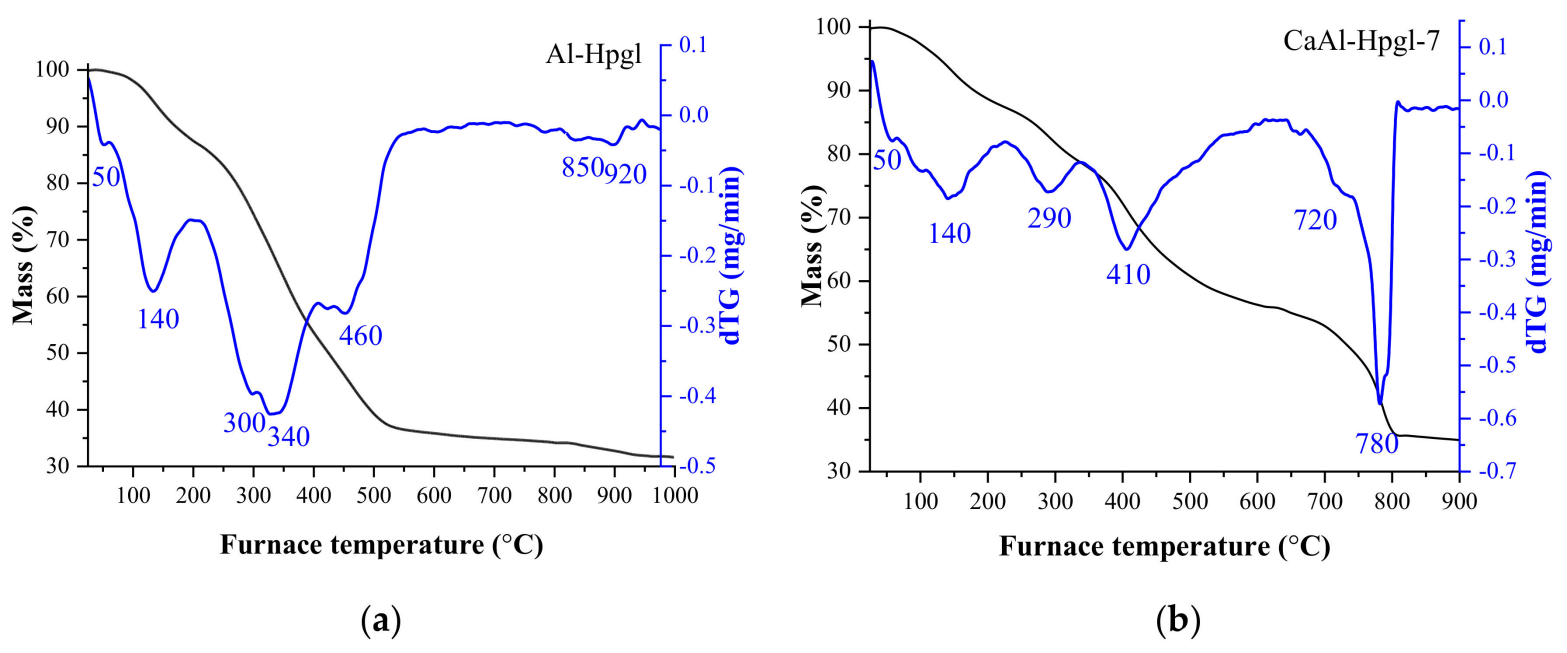
Thermograms (left axis, black solid line) and differential thermograms (right axis, blue solid line) of the Al-binary (**a**) and a representative ternary (CaAl-Hpgl-7) compound (**b**).

The thermograms of both the Al- and Ca-Hpgl solids (Figure 6a and Figure S4) as well as the CaAl-Hpgl-7 ternary compound (Figure 6b) exhibit well-distinguishable mass loss processes. For all compounds, the physically adsorbed water (i.e., the fraction of water, which is bound to the surface) evaporate first at 50 °C, followed by the release of lattice water below ~180 °C.

**Figure 7:**
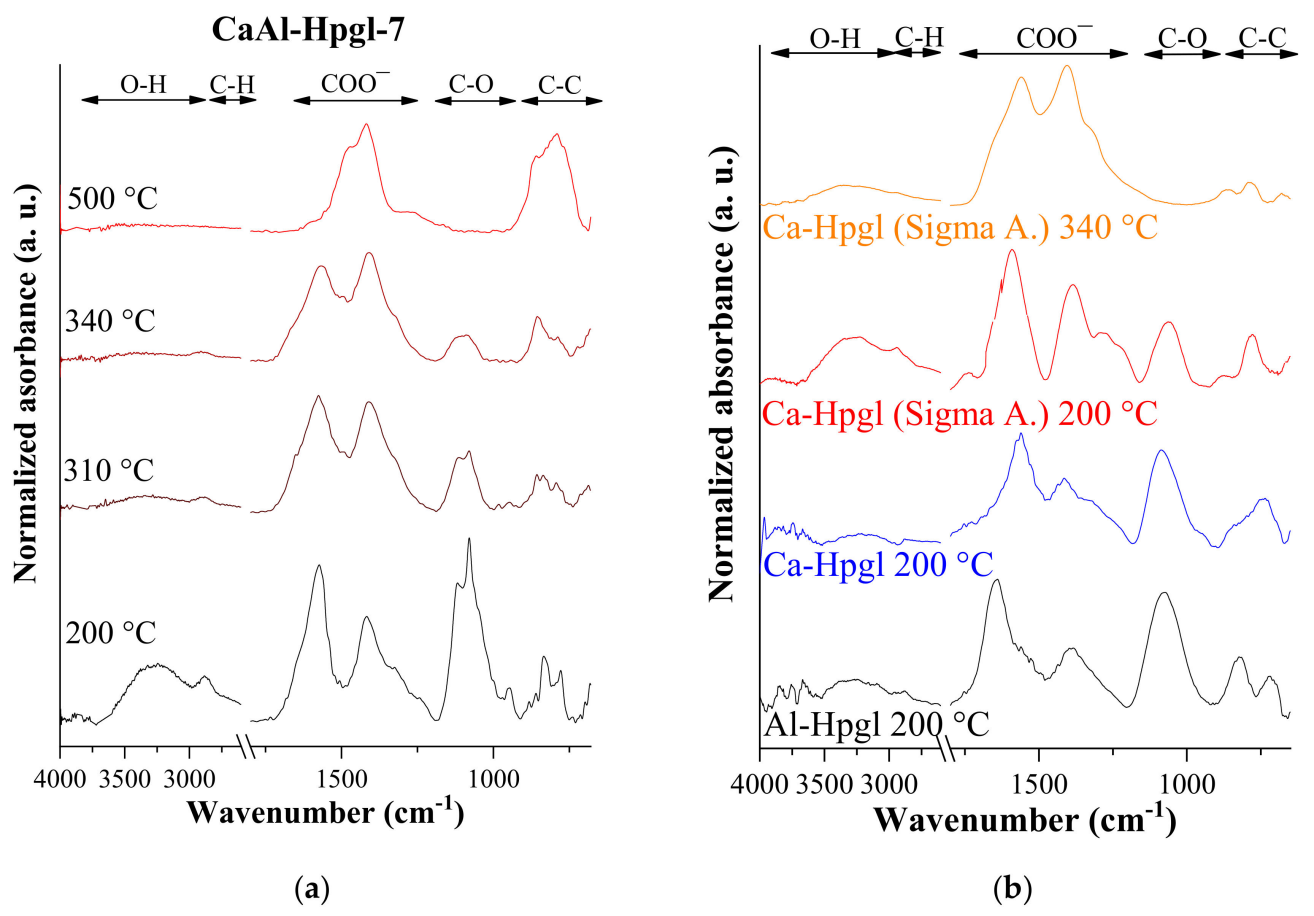
Infrared spectra of the CaAl-Hpgl-7 ternary solid compound after heat treatment at different temperatures (**a**), and the binary and commercial solids at 200 °C and 340 °C (**b**).

The next major loss peak appears between 180 and 400 °C (Al-Hpgl) or 180 and 350 °C (Ca-Hpgl and CaAl-Hpgl-5). To reveal the underlying chemical processes, infrared spectra of the CaAl-Hpgl-7 solid calcined at 200, 310, 340 and 500 °C were recorded (Figure 7a). The sample calcined at 200 °C remains essentially intact, since the peaks in the corresponding spectrum are in similar positions and of similar shape as compared to the initial solid (Figure 3, right). However, above 310 °C, marked variations can be observed: the intensities of the Hpgl O–H/C–H stretching vibrational modes at 2750–3750 cm^−1^ gradually decrease in conjunction with the weakening of the C–O bands between 900 and 1200 cm^−1^. Parallel to the mineralization of the heptagluconate, dehydration of the Al(OH)_x_^(3-x)+^ ion may also occur, since this step takes place at < 300 °C in the case of Al(OH)_3_ [28].

Between 340 and 500 °C, the caramelization of the sugar proceeds with similar mass losses (16–22%), presumably along with the dehydration of the Ca(OH)_y_^(2−y)+^ (which occurs at ~460 °C, see the TG curve of Ca(OH)_2_ in Figure S6). The IR spectrum of the solids calcined at 500 °C shows the disappearance of the C–H and C–O bonds. The collapse of the heptagluconate moiety is also shown by the variation of asymmetric and symmetric vibrations of the COO^−^ groups (at 1570 and 1410 cm^−1^). Meanwhile, a signal at 1420 cm^−1^ ascribed to the surface-adsorbed CO_2_ molecules as well as a resonance mode at 1480 cm^−1^ corresponding to the in situ generated calcite phase appeared [29].

Above 500 °C, the merger of calcite phase decomposition (Figure S6) with the final-step mineralization of Hpgl^−^ could be observed; the decarbonization processes took place in the most substantial amounts (24–30% mass losses). The caramelization and mineralization processes were confirmed by the XRD measurements shown in Figure S7. A notable feature of the Al-Hpgl and Ca-Hpgl binary samples is that the decomposition of Hpgl^−^ commenced at temperatures higher than those in the commercially available sodium and calcium salts of heptagluconate. The ternary samples exhibited even more pronounced thermal stabilities: for the binary solids heat-treated at 200 °C, a significant decrease in the intensities of their C–O peaks (900–1200 cm^−1^) on their infrared spectra could be observed (Figure 6), while those of the ternary compounds were unscathed. This enhanced thermal stability of Hpgl^−^ is more evident for the calcination of samples at 340 °C: the C–O bands and even the O–H/C–H stretching modes (2750–3750 cm^−1^) of the Na- and Ca-salts of heptagluconate disappeared from the spectrum, while these peaks were still observable for CaAl-Hpgl-7 after heat-treatment at 340 °C. Moreover, the calculated mass losses remained under ~13% for the ternary solids between 200 and 350 °C, and these values were significantly larger (21 and 34%) for the binary precipitates (Table S2.). Ultimately, these observations indicate that the ternary compounds are more stable than the binary ones suggesting that the simultaneous binding of Ca^2+^ and Al^3+^ yields overall stronger metal-sugar interactions.

## 3. Materials and Methods

### 3.1. Reagents and Solutions

All solutions were prepared using deionized water (Merck Millipore Milli–Q^®^, Burlington, MA, USA). Stock solutions were prepared using sodium α-d-heptagluconate (Sigma-Aldrich, St. Louis, MO, USA, ≥99% purity), calcium α-d-heptagluconate dihydrate (Sigma–Aldrich, ≥98.0% purity), calcium chloride dihydrate (Analar Normapur, supplier VWR Hungary, Hungary, a.r. grade), magnesium sulfate heptahydrate (Analar Normapur, a.r. grade) and hydrochloric acid (Scharlau, Barcelona, Spain, a.r. grade). The ionic strength of the solutions was set with sodium chloride (VWR, Radnor, PA, USA, a.r. grade) to 4.0 M. Sodium α-d-heptagluconate was purchased as non-stoichiometrically hydrated salt; therefore, its water content was determined by weighting the solid before and after heating it at 80 °C for six hours.

The HCl stock solutions (~1.0 M) were made by the volumetric dilution of 37 *w*/*w*% HCl solution and were standardized with KHCO_3_ solution. Sodium hydroxide solutions were gravimetrically diluted from a concentrated (~50 *w*/*w*%), carbonate-free NaOH solution, as previously described [30]. Sodium aluminate solutions (~4.0 M NaAl(OH)_4_, ~4.0 M NaOH) were prepared by dissolving high purity aluminum wires (J.M. & Co., Brackley, UK, 99.99% purity) in a carbonate free NaOH solution, following the steps previously described [31].

The concentration of CaCl_2_ and NaAl(OH)_4_ solutions were determined by EDTA titration prior to the measurements.

### 3.2. Synthesis and Preparation of the Samples

The binary calcium- or aluminum-containing solids were precipitated from solutions comprising [Hpgl^−^]_T_ = 0.200 M, [Ca^2+^]_T_ = 0.135 M, [OH^−^]_T_ = 0.250 M and [Hpgl^−^]_T_ = 0.146 M, [Al(OH)^−^]_T_ = 0.292 M, [OH^−^]_T_ = 0.146 M, respectively (herefter, subscript T denotes total or analytical concentrations). As for the ternary solution, solid samples were obtained by gradually adding 0.9923 M CaCl_2_ (I = 4 M NaCl) to solutions containing aluminate and heptagluconate ions at various amounts. The initial concentrations of different components were varied between [Hpgl^−^]_T_ = 0.100–0.500 M, [Al(OH)^−^]_T_ = 0.100–0.375 M and [OH^−^]_T_ = 0.050–0.250 M. The concentration of calcium(II) in the solutions before the appearance of the precipitate ranged from 0.06 M to 0.32 M. The sample identifiers and the corresponding solution compositions at the onset of precipitation are listed in Table 2.

All samples were filtered through a hydrophilic PVDF 0.45 μm porous size membrane filter and rinsed with deionized water to remove NaCl traces from the surface. Prior to measurements, the solids were kept in a desiccator.

### 3.3. Methods of Structural Characterization

The solid compounds were first characterized by a Rigaku MiniFlex Type II. X-ray diffractometer (Tokyo, Japan) applying CuKα radiation (λ = 0.15406 nm). The diffractograms were recorded in a 2θ° range of 5–65° with 3°·min^−1^ scanning speed.

The morphologies of the binary and ternary precipitates were visualized by scanning electron microscopy (SEM, Hitachi S-4700, Chiyoda City, Tokyo, Japan) at various magnifications and acceleration voltages. The segregation of the elements in the solids was analyzed by an energy-dispersive X-ray spectroscopy accessory connected to the scanning electron microscope (EDX, Röntec QX2 spectrometer equipped with Be window, Berlin, Germany). The thermal properties of the samples were investigated by a Setaram Labsys derivatograph (Setaram Hungary, Hungary) operating under a constant flow of air at 5 °C·min^−1^ heating rate. For the analysis, 25–30 mg portions of the solids were placed into high-purity alpha-alumina crucibles.

A Bio-Rad Digilab Division FTS65A/896 FT-IR Spectrometer (California, CA, USA) with a Harrick’s Meridian™ SplitPea Single Reflection Diamond ATR Accessory (Cambridge, MA, USA) was used to record the spectra of the samples at room temperature. The measurements were performed in the range of 4000–400 cm^−1^ at 4 cm^−1^ optical resolution and 128 interferograms were averaged to achieve good signal to noise ratio. Raman spectra were recorded in the range of 3500 cm^−1^–100 cm^−1^ at 2 cm^−1^ resolution by a Thermo Scientific Raman DXR microscope (Waltham, MA, USA) at room temperature. The light source was a diode-pumped, frequency-doubled Nd:YAG laser (λ = 760 nm), which was used at 15 mW maximum laser power.

Spectra were analyzed (including deconvolution, peak fitting, etc.) with the aid of the Thermo Galactic Inc. GRAMS/AI version 7 software (Waltham, MA, USA) [32].

The total concentrations of Ca^2+^ and Al^3+^ ions were determined with a Thermo Scientific iCAP 7400 ICP-OES DUO spectrometer with radial plasma viewing. An accurately weighed ≈ 25 mg portion of each sample was dissolved in 5 cm^3^ ≈ 2 M hydrochloric acid and diluted with deionized water. The calibration was performed using an ICP Multielement standard solution IV (CertiPUR^®^, supplier VWR Hungary, Hungary) along with internal magnesium standard.

The spectra of solutions containing heptagluconate were recorded with a Specord 210 plus double beam UV-Vis spectrophotometer Germany) at (25.1 ± 0.1) °C, in the wavelength range of 185–500 nm with 0.1 nm resolution. The optical path length of the quartz cuvette was 1 cm. Prior to the measurements, a known amount of each sample (≈50 mg) was dissolved in ≈ 2 M HCl. Due to the strongly acidic medium, Hpgl^−^ undergoes lactonization, a reaction in which cyclic esters are formed [16], giving rise to a gradual shift in the absorbance. To minimize this effect, all samples were measured right after their preparation. Additionally, the absorbance readings were performed at the isosbestic point at 218 nm, where they are invariant of the progress of lactonization (Figure S8). The calculation of concentrations in the samples was based on the calibration curve recorded for a solution series with known concentrations of Hpgl^−^ (0.001 M–0.010 M) at ≈2 M HCl (Figure S9), applying Beer’s law.

## 4. Conclusions

In this work, the solid phases obtained from solutions containing Ca^2+^, Al^3+^ and Hpgl^−^ ions, were characterized with various experimental methods. These compounds were found to have a low degree of crystallinity, homogeneous elemental distribution and a significantly different morphology than those of the commercially available Hpgl^−^ salts, which is in line with the results published by Kim and Lee [22].

Based on the results of spectrophotometry and elemental analysis, two types of compounds could be distinguished: CaAlHpgl(OH)_4_^0^ along with Ca_2_AlHpgl_2_(OH)_5_^0^ forms with n_OH/_n_Hpgl_ > 1.0, while for n_OH/_n_Hpgl_ < 1.0, the more uniform Ca_3_Al_2_Hpgl_3_(OH)_9_^0^ stoichiometry can be proposed. The latter strongly resembles the composition of Ca_3_Al_2_(OH)_12_ (Tricalcium aluminate), which may explain the similar IR spectra observed for the reaction of TCA with Gluc^−^ by others earlier [23]. Regarding the binary compounds, the formation of Al_3_Hpgl(OH)_8_^0^ and varius mixed Ca-containing binary compounds was invoked.

FT-IR and Raman spectroscopic measurements reinforced the assumption that the solid compounds can be divided into two groups: the different peak positions of the fitted carboxylate bands, based on the results of Fourier self-deconvolution, may refer to the coordination of Ca^2+^ or Al^3+^ ions. The ratio of integrated intensities for these peaks clearly marks the boundary between the two groups of compounds. Furthermore, the centrally symmetric arrangement of Hpgl around the metal ion was inferred from the positions of the fitted antisymmetric carboxylate stretching bands.

Based on the thermal analysis of the commercially available salts of heptagluconate along with the binary and ternary compounds, an increased stability of the binary and ternary compounds was observed. This stability was the highest in case of ternary compounds, which was attributed to the simultaneous binding of both metal ions to the Hpgl.

## Figures and Tables

**Table 1:** *w*/*w*% distribution of the components and the calculated cumulative stoichiometry of the corresponding solids.

Sample ID	Ca^2+^ (*w*/*w*%)	Al^3+^ (*w*/*w*%)	Hpgl^−^ (*w*/*w*%)	Calculated Cumulative Stochiometry
Ca-Hpgl	7.6	-	74.9	CaHpgl_1.8_(OH)_0.3_ × 4.9 H_2_O
Al-Hpgl	-	17.0	60.5	AlHpgl_0.4_(OH)_2.6_
CaAl-Hpgl-1	16.7	5.7	58.0	Ca_2_AlHpgl_1.2_(OH)_5.7_
CaAl-Hpgl-2	13.4	3.3	67.9	Ca_2.8_AlHpgl_2.5_(OH)_6.0_ × 0.5 H_2_O
CaAl-Hpgl-3	12.6	4.1	66.6	Ca_2.1_AlHpgl_1.9_(OH)_5.2_ × 0.6 H_2_O
CaAl-Hpgl-4	14.8	2.9	62.3	Ca_3.5_AlHpgl_2.6_(OH)_7.4_ × H_2_O
CaAl-Hpgl-5	11.6	4.4	63.9	Ca_1.8_AlHpgl_1.8_(OH)_4.8_ × 1.3 H_2_O
CaAl-Hpgl-6	12.4	5.7	64.9	Ca_1.5_AlHpgl_1.4_(OH)_4.6_ × 0.1 H_2_O
CaAl-Hpgl-7	13.4	6.8	61.3	Ca_1.3_AlHpgl_1.2_(OH)_4.5_ × 0.6 H_2_O
CaAl-Hpgl-8	12.0	5.4	63.9	Ca_1.5_AlHpgl_1.4_(OH)_4.6_ × 0.6 H_2_O

**Table 2:** The list of the as-prepared solid precipitates and the analytical concentrations.

Sample ID	[Ca^2+^]_T_/M	[Al(OH)^−^]_T_/M	[Hpgl^−^]_T_/M	[OH^−^]_T_/M
Ca-Hpgl	0.135	-	0.200	0.250
Al-Hpgl	-	0.292	0.146	0.146
CaAl-Hpgl-1	0.055	0.095	0.095	0.236
CaAl-Hpgl-2	0.120	0.088	0.176	0.220
CaAl-Hpgl-3	0.122	0.132	0.175	0.220
CaAl-Hpgl-4	0.063	0.047	0.094	0.235
CaAl-Hpgl-5	0.322	0.169	0.338	0.169
CaAl-Hpgl-6	0.197	0.081	0.160	0.042
CaAl-Hpgl-7	0.200	0.120	0.160	0.042
CaAl-Hpgl-8	0.175	0.165	0.165	0.044

## Supplementary Materials

The following are available online, Figure S1: X-ray diffractograms of the ternary precipitates. Figure S2: Metal distribution visualization of the CaAl-Hpgl-5. Yellow dots: aluminum, red dots: calcium. Figure S3: The observable difference between the O-H stretching range and in that of the COO^−^ group, presented on the infrared and Raman spectrum of sample CaAl-Hpgl-3. Figure S4: Fourier Self-Deconvolution performed on the IR and Raman spectra of sample CaAl-Hpgl-8. Figure S5: Thermograms of the commercial heptagluconic acid sodium and calcium salts from Sigma. Aldrich. Figure S6: Thermograms of the commercial calcium hydroxide and carbonate salts from Sigma Aldrich. Figure S7. XRD diffractograms of the heat-treated CaAl-Hpgl-7 sample. The inorganic CaO and Al_2_O_3_ compound were identified, which supports the suggested mineralization process. Figure S8: Time-dependent UV-Vis spectra of heptagluconic acid. Total concentrations: [NaHpgl]_T_ = 0.005 M, [HCl]_T_ = 2.0075 M (t = 25.0 ± 0.1 °C). Figure S9: Calibration curve of heptagluconic acid. The result of linear fitting is depicted as dashed line. Table S1: Results of the peak fitting, performed on the carboxylate region (1850–1200 cm^−1^) of the IR (plain background) and Raman (grey background) spectra. Table S2: Mass losses of the solids at different temperature ranges.

## References

[1] Roach G.I. (2016). The equilibrium approach to causticisation for optimising liquor causticisity. Essential Readings in Light Metals.

[2] Fischer R., Kuzel H.-J. (1982). Reinvestigation of the system C_4_A.nH_2_O–C_4_A.CO_2_.nH_2_O. Cem. Concr. Res..

[3] Malts N. (1992). Efficiency of lime use in Bayer alumina production. Light Metals.

[4] Whittington B. (1996). The chemistry of CaO and Ca (OH)_2_ relating to the Bayer process. Hydrometallurgy.

[5] Terzis A., Filippakis S., Kuzel H.-J., Burzlaff H. (1987). The crystal structure of Ca_2_Al(OH)_6_Cl.2H_2_O. Z. Kristallogr. Cryst. Mater..

[6] Rosenberg S.P., Wilson D.J., Heath C.A. (2016). Some aspects of calcium chemistry in the Bayer process. Essential Readings in Light Metals.

[7] The P.J., Sivakumar T.J. (1985). The effect of impurities on calcium in Bayer liquor. Essential Readings in Light Metals.

[8] Singh N.B. (1976). Effect of gluconates on the hydration of cement. Cem. Concr. Res..

[9] Motekaitis R.J., Martell A.E. (1984). Complexes of aluminum (III) with hydroxy carboxylic acids. Inorg. Chem..

[10] Tajmir-Riahi H.A. (1990). Carbohydrate complexes with alkaline earth metal ions. Interaction of D-glucono-1,5-lactone with the Mg(II), Ca(II), Sr(II), and Ba(II) cations in the crystalline solid and aqueous Solution. J. Inorg. Biochem..

[11] Best W.M., Harrowfield J.M., Shand T.M., Stick R.V. (1994). Aluminum (III) coordination to hydroxy carboxylates: The influence of hydroxy substituents enabling tridentate binding. Aust. J. Chem..

[12] Lakatos A., Kiss T., Bertani R., Venzo A., Di Marco V.B. (2008). Complexes of Al (III) with d-gluconic acid. Polyhedron.

[13] Kieboom A.P.G., Buurmans H.M.A., van Leeuwen L.K., van Benschop H.J. (2010). Stability constants of (hydroxy)carboxylate- and alditol-calcium(II) complexes in aqueous medium as determined by a solubility method. Recl. Trav. Chim. Pays-Bas.

[14] Vavrusova M., Liang R., Skibsted L.H. (2014). Thermodynamics of Dissolution of Calcium Hydroxycarboxylates in Water. J. Agric. Food Chem..

[15] Pallagi A., Tasi Á.G., Peintler G., Forgo P., Pálinkó I., Sipos P. (2013). Complexation of Al(iii) with gluconate in alkaline to hyperalkaline solutions: Formation, stability and structure. Dalton Trans..

[16] Buckó Á., Kutus B., Peintler G., Kele Z., Pálinkó I., Sipos P. (2020). Stability and structural aspects of complexes forming between aluminum (III) and D-heptagluconate in acidic to strongly alkaline media: An unexpected diversity. J. Mol. Liq..

[17] Kutus B., Gaona X., Pallagi A., Pálinkó I., Altmaier M., Sipos P. (2020). Recent advances in the aqueous chemistry of the calcium(II)-gluconate system—Equilibria, structure and composition of the complexes forming in neutral and in alkaline solutions. Coord. Chem. Rev..

[18] Venema F., Peters J., Van Bekkum H. (1993). Multinuclear-magnetic-resonance study of the coordination of aluminium (III)-aldarate complexes with calcium (II) in aqueous solution. Recl. Trav. Chim. Pays-Bas.

[19] Bechtold T., Burtscher E., Turcanu A. (2002). Ca^2+^–Fe^3+^–D-gluconate-complexes in alkaline solution. Complex stabilities and electrochemical properties. J. Chem. Soc. Dalton Trans..

[20] Tits J., Wieland E., Bradbury M.H. (2005). The effect of isosaccharinic acid and gluconic acid on the retention of Eu(III), Am(III) and Th(IV) by calcite. Appl. Geochem..

[21] Gaona X., Montoya V., Colàs E., Grivé M., Duro L. (2008). Review of the complexation of tetravalent actinides by ISA and gluconate under alkaline to hyperalkaline conditions. J. Contam. Hydrol..

[22] Kim C.-E., Lee S.-H. (1986). Effect of Sodium Gluconate on the Hydration of Tricalcium Aluminate(II) Early Hydration Behavior. J. Korean Ceram. Soc..

[23] Kim C.-E., Lee S.-H., Lee S.-K. (1990). Complex formation between 3CaO.Al_2_O_3_ and Sodium Gluconate. J. Korean Ceram. Soc..

[24] Colthup N., Daly L.H., Wiberley S.E. (1990). Introduction to Infrared and Raman Spectroscopy.

[25] Deacon G., Phillips R. (1980). Relationships between the carbon-oxygen stretching frequencies of carboxylato complexes and the type of carboxylate coordination. Coord. Chem. Rev..

[26] Nakamoto K. (2006). Infrared and Raman Spectra of Inorganic and Coordination Compounds. Handbook of Vibrational Spectroscopy.

[27] Papageorgiou S.K., Kouvelos E.P., Favvas E.P., Sapalidis A.A., Romanos G.E., Katsaros F.K. (2010). Metal–carboxylate interactions in metal–alginate complexes studied with FTIR spectroscopy. Carbohydr. Res..

[28] Yang W., Kim Y., Liu P.K.T., Sahimi M., Tsotsis T.T. (2002). A study by in situ techniques of the thermal evolution of the structure of a Mg–Al–CO_3_ layered double hydroxide. Chem. Eng. Sci..

[29] Frost R.L., Palmer S.J., Theiss F. (2011). Synthesis and Raman spectroscopic characterisation of hydrotalcites based on the formula Ca_6_Al_2_(CO_3_)(OH)_16._4H_2_O. J. Raman Spectrosc..

[30] Sipos P., May P.M., Hefter G.T. (2000). Carbonate removal from concentrated hydroxide solutions. Analyst.

[31] Sipos P., Capewell S.G., May P.M., Hefter G., Laurenczy G., Lukács F., Roulet R. (1998). Spectroscopic studies of the chemical speciation in concentrated alkaline aluminate solutions. J. Chem. Soc. Dalton Trans..

[32] (2004). Thermo Scientific^™^ GRAMS/AI^™^ Version 7.

