# Supplementary material for: The Structure and Thermal Properties of Solid Ternary Compounds Forming with Ca2+, Al3+ and Heptagluconate Ions
Source: Molecules. 2020 Oct 14;25(20):4715. doi: 10.3390/molecules25204715 (PMC7587368; doi:10.3390/molecules25204715)
Supplement: Supplementary file 1 [file molecules-25-04715-s001.pdf]

molecules

Electronic supplementary information

The Structure and Thermal Properties of Solid Ternary Compounds Forming with Ca^{2+}, Al^{3+} and Heptagluconate Ions

Ákos Buckó[1], Zsolt Kása[1], Márton Szabados[2], Bence Kutus[3], Ottó Berkesi[4], Zoltán Kónya[5], Ákos Kukovecz[5], Pál Sipos[1] and István Pálinkó[2,*]

[1] Department of Inorganic and Analytical Chemistry, University of Szeged, Dóm sqr. 7, Szeged, H-6720, Hungary
[2] Department of Organic Chemistry, University of Szeged, Dóm sqr. 8, H-6720 Szeged, Hungary
[3] Department of Molecular Spectroscopy, Max Planck Institute for Polymer Research, D-55128 Mainz, Germany
[4] Department of Physical Chemistry and Materials Science, University of Szeged, Rerrich B. sqr. 1, H-6720 Szeged, Hungary
[5] Department of Applied and Environmental Chemistry, University of Szeged, Rerrich B. sqr. 1, H-6720 Szeged, Hungary

* Correspondence: palinko@chem.u-szeged.hu; Tel.: +36-62-544-288

S1 Crystallinity and morphology of the ternary samples

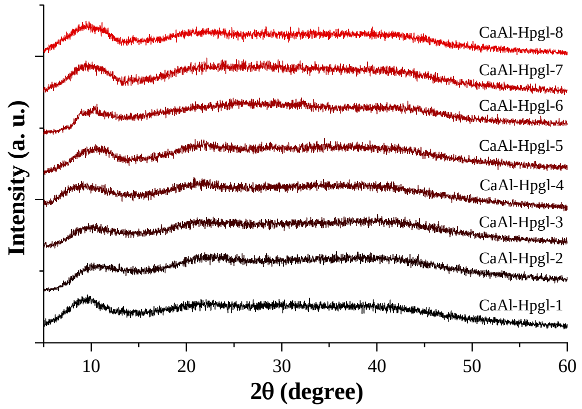

Figure S1 X-ray diffractograms of the ternary precipitates.

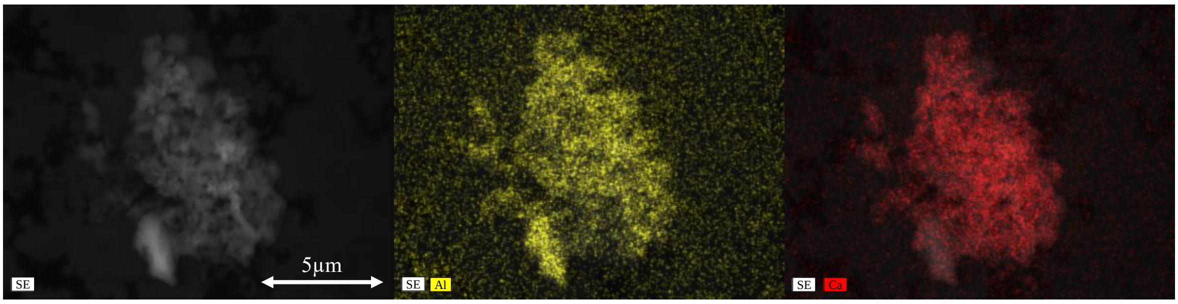

Figure S2 Metal distribution visualization of the CaAl-Hpgl-5. Yellow dots: aluminum, red dots: calcium.

S2 The effect of metal coordination on the infrared and Raman spectra of sodium heptagluconate

The information provided by infrared and Raman spectra of the same sample complement each other, since the general selection rules differ from each other. The infrared intensities are proportional to the square of the transitional dipole moment, while the Raman intensities are determined by the change in the polarizability tensor during the transition.

The vibrational normal modes of the D-heptagluconate ion can be classified from that point of view. The normal modes dominated by the displacement of the hydrogen atoms in the O-H groups produce high transitional dipole moment, but hardly influence the polarizability tensor of the molecule. Their intensities are high in the absorption spectrum, and they are practically invisible in the Raman spectrum.

On the other hand, the normal modes, dominated by the combinations of the stretchings of C-C bonds alter the polarizability tensor, but produce negligible change in the dipole moment. So, their intensities are low in the infrared and relatively high in the Raman spectrum [1].

The normal modes, dominated by the carboxylate group, have both effects, since the delocalized 4π electron system strongly shifted to the direction of the oxygen atoms, but prone to further polarization, so can be seen in both spectra. According to the above considerations, the infrared spectra of the samples, provide information mainly on the coordination properties of the sugar, while the Raman spectra on the conformation of the backbone.

This difference is most obvious in the O-H stretching range and in that of the carboxylate group. It is shown in **Figure (a) and (b)**, in the case of sample CaAl-Hpgl-3.

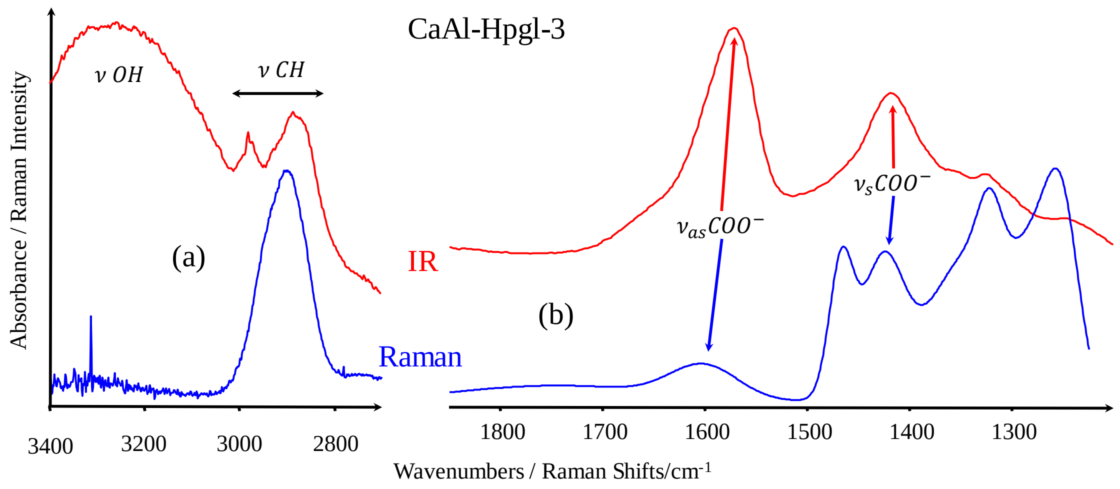

Figure S3 The observable difference between the O-H stretching range and in that of the COO– group, presented on the infrared and Raman spectrum of sample CaAl-Hpgl-3.

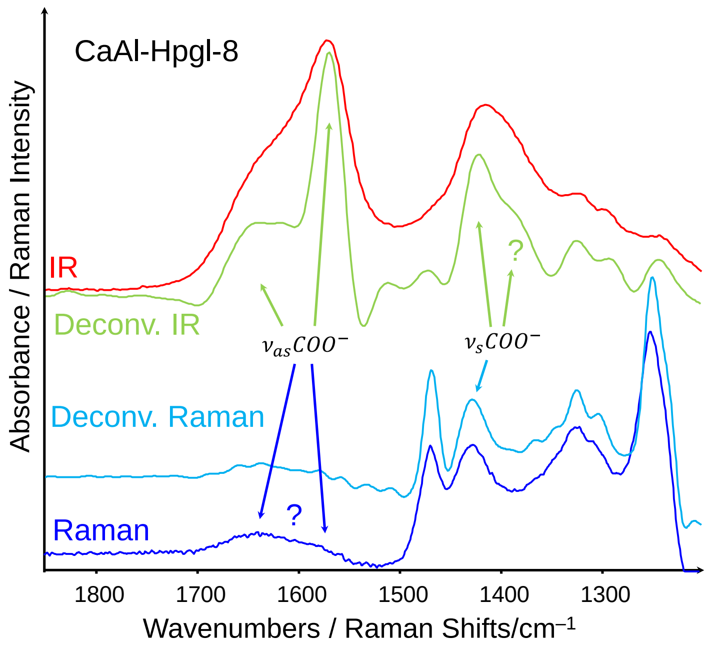

Figure S4 Fourier Self-Deconvolution performed on the IR and Raman spectra of sample CaAl-Hpgl-8

Peak fitting was performed with every spectrum. The best was achieved by the following strategy. The initial number of fitted peaks and their relative intensities were determined by the result of the Fourier Self-Deconvolution. Gaussian functions were fitted in the first round. The residual curve were examined and the number of peaks was reduced when less peaks seemed to be appropriate to describe the fine structure of the spectrum. When the residual curve contained obvious peak-like features and it was impossible to describe the fine structure of the spectrum further peaks were added accordingly. The fitted functions were changed to mixture of Gaussian and Lorentzian functions, and their ratios were also fitted in the final round. The process was finished when the residual curve showed noise-like character.

Table S1 Results of the peak fitting, performed on the carboxylate region (1850 – 1200 cm^{-1}) of the IR (plain background) and Raman (grey background) spectra

Sample ID	ν /cm^{-1}	Lorentz %	$\frac{Area_{n+1}}{Area_n}$ /%	ν /cm^{-1}	Lorentz %	$\frac{Area_{n+1}}{Area_n}$ /%	ν /cm^{-1}	Lorentz %	$\frac{Area_{n+1}}{Area_n}$ /%
Ca-Hpgl	–	–	–	1590	0.63	0.4	1413	0.00	3.3
	1609	0.00	–	–	–	–	1428	0.00	1.1
Al-Hpgl	1634	0.00	–	1554	0.21	2.2	1393	0.47	2.4
	1650	0.31	0.5	–	–	–	1405	0.00	2.0
CaAl-Hpgl-1	1621	0.27	1.9	1569	0.25	1.6	1418	1.00	2.0
	1600	0.00	–	–	–		1427	0.00	1.5
CaAl-Hpgl-2	1609	0.01	1.3	1574	0.04	1.7	1419	0.81	2.0
	1601	0.00	–	–	–		1426	0.00	1.4
CaAl-Hpgl-3	1614	0.01	1.6	1571	0.01	1.4	1419	0.86	2.1
	1602	0.00	–	–	–		1427	0.00	1.4
CaAl-Hpgl-4	1613	0.34	1.2	1574	0.01	1.6	1417	0.64	2.1
	1603	0.00	–	–	–		1426	0.00	1.7
CaAl-Hpgl-5	1604	0.13	0.8	1568	0.34	1.7	1419	1.00	2.3
	1631	0.00	0.4	1581	0.00		1429	0.00	1.7
CaAl-Hpgl-6	1625	0.34	1.7	1565	0.60	0.6	1426	1.00	3.6
	1631	0.00	0.2	1576	0.00	8.9	1429	0.21	1.6
CaAl-Hpgl-7	1619	0.00	0.6	1569	0.00	1.0	1423	0.50	3.5
	1634	0.00	0.2	1576	0.00	8.8	1429	0.33	1.7
CaAl-Hpgl-8	1621	0.01	0.7	1569	0.39	0.8	1423	0.96	4.0
	1634	0.00	0.2	1576	0.00	10.6	1429	0.33	1.6

S3 Thermal analysis

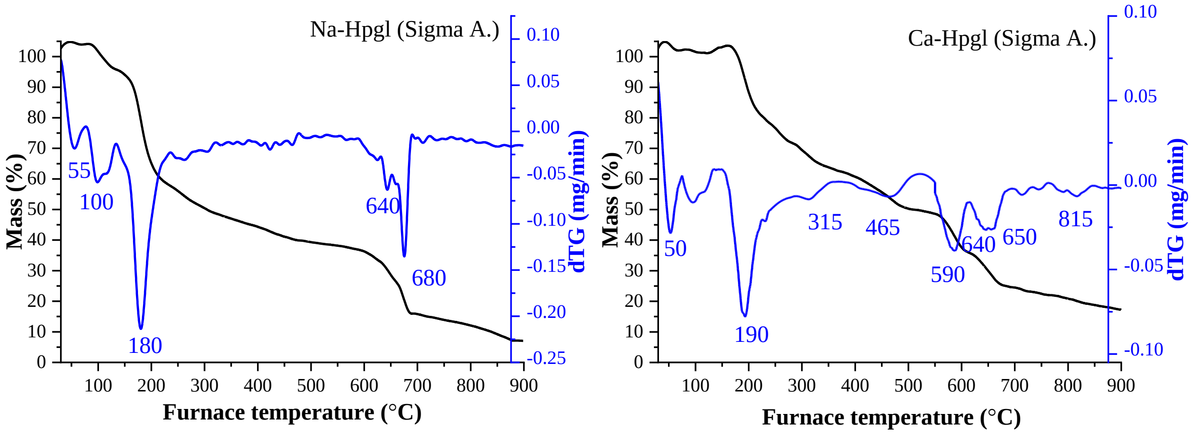

Figure S5. Thermograms of the commercial heptagluconic acid sodium-, and calcium salts from Sigma. Aldrich.

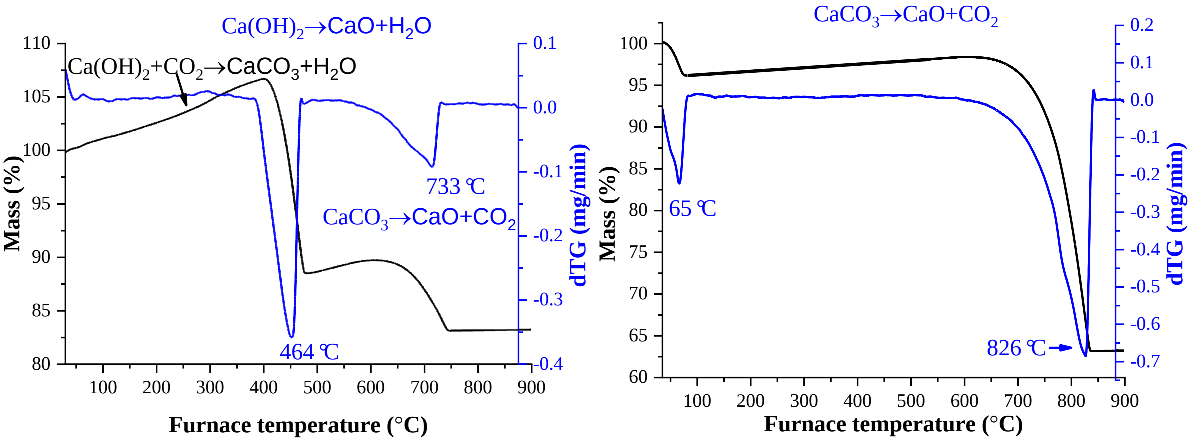

Figure S6. Thermograms of the commercial heptagluconate sodium-, and calcium salts from Sigma. Aldrich.

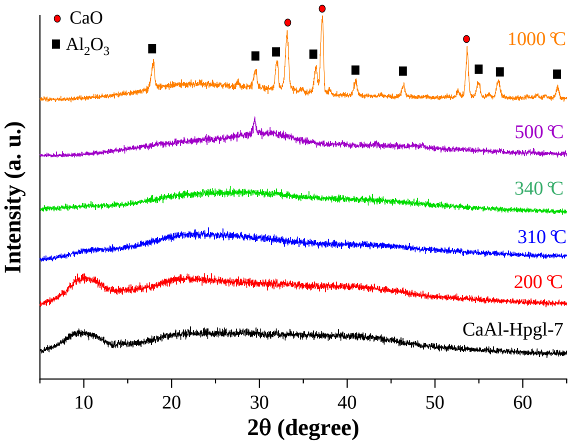

Figure S7. XRD diffractograms of the heat-treated CaAl-Hpgl-7 sample. The inorganic CaO and Al_2O_3 compounds were identified, consequently, the mineralization process was confirmed.

Table S2 Mass losses of the solids at different temperature ranges.

Sample ID	Mass loss under 200°C (%)	Mass loss 200–350°C (%)	Mass loss 350–500°C (%)	Mass loss above 500°C (%)
Ca-Hpgl	9	20.9	22.1	24.6
Al-Hpgl	12.5	33.8	17.1	4.9
CaAl-Hpgl-1	14.9	7.4	15.8	26.6
CaAl-Hpgl-2	13.2	12.6	15.8	29.7
CaAl-Hpgl-3	12.7	11.0	16.3	28.4
CaAl-Hpgl-4	13.5	10.6	14.4	29.7
CaAl-Hpgl-5	10.5	12.0	17.2	29.5
CaAl-Hpgl-6	11.3	10.9	17.0	26.2
CaAl-Hpgl-7	12.7	10.8	17.4	27.0
CaAl-Hpgl-8	14.7	11.0	16.8	24.9

S4 UV-Vis spectroscopy

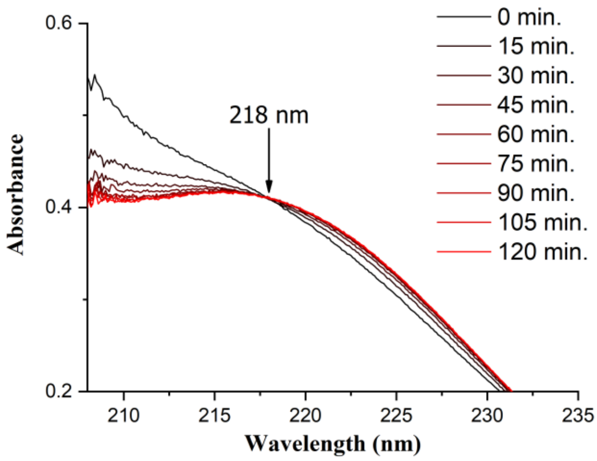

Figure S8 Time-dependent UV-Vis spectra of heptagluconic acid. Total concentrations: $[NaHpgl]_T$ = 0.005 M, $[HCl]_T$ = 2.0075 M (t = 25.0 ±0.1°C).

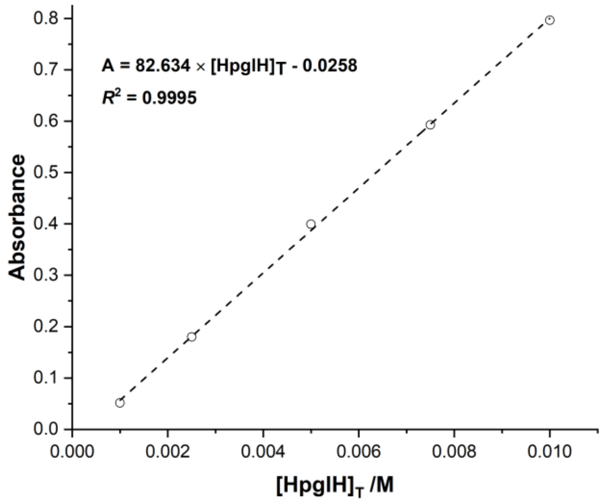

Figure S9 Calibration curve of heptagluconic acid. The result of linear fitting is depicted as dashed line.

References

1. Larkin, P. *Infrared and Raman spectroscopy: principles and spectral interpretation*; Elsevier, 2017; ISBN 0-12-804209-5.
